# Proteomic profiling of thyroid tissue in patients with obesity and benign diffuse goiter

**DOI:** 10.3389/fendo.2022.923465

**Published:** 2022-07-28

**Authors:** Hicham Benabdelkamel, Mohamed Rafiullah, Afshan Masood, Abdulaziz Alsaif, Mohthash Musambil, Assim A. Alfadda

**Affiliations:** ^1^ Proteomics Resource Unit, Obesity Research Center, College of Medicine, King Saud University, Riyadh, Saudi Arabia; ^2^ Strategic Center for Diabetes Research, College of Medicine, King Saud University, Riyadh, Saudi Arabia; ^3^ Division of Endocrine and Breast Surgery, Department of Surgery, College of Medicine and King Saud University Medical City, King Saud University, Riyadh, Saudi Arabia; ^4^ Department of Medicine, College of Medicine and King Saud University Medical City, King Saud University, Riyadh, Saudi Arabia

**Keywords:** thyroid tissue, goiter, proteomics, obese, 2D-DIGE, MALDI-TOF

## Abstract

Goiter is a term to describe the enlargement of the thyroid gland. The pathophysiology and molecular changes behind development of diffuse benign goiter remains unclear. The present study targeted to identify and describe the alterations in the thyroid tissue proteome from patients (obese euthyroid) with benign diffuse goiter (BDG) using proteomics approach. Thyroid tissue samples, from 7 age and sex matched, patients with BDG and 7 controls were obtained at the time of surgery. An untargeted proteomic analysis of the thyroid tissue was performed out utilizing two-dimensional difference (2D-DIGE) in gel electrophoresis followed by matrix-assisted laser desorption/ionization time-of-flight mass spectrometry (MALDI-TOF-MS) for identification of the proteins. Progenesis software was used to identify changes in expression of tissue proteins and found statistically significant differences in abundance in a total of 90 proteins, 46 up and 44 down (1.5-fold change, ANOVA, p ≤ 0.05) in BDG compared to the control group. Bioinformatic analysis using Ingenuity Pathway Analysis (IPA) identified dysregulation of signalling pathways linked to ERK1/2, Glutathione peroxidase and NADPH oxidase associated to organismal injury and abnormalities, endocrine system disorders and cancer. The thyroid tissue proteome in patients with BDG revealed a significant decrease in thyroglobulin along with dysregulation of glycolysis and an increase in prooxidant peroxidase enzymes. Dysregulation of metabolic pathways related to glycolysis, redox proteins, and the proteins associated with maintaining the cytoskeletal structure of the thyrocytes was also identified.

## Introduction

The term goiter generally describes a thyroid gland enlargement. It presents usually as an incidental swelling in the neck discovered by the patient or on routine physical examination. Various causes ranging from physiological to pathological mechanisms lead to hypertrophy of thyroid gland. Physiological increase in the thyroid gland volume is seen during adolescence, pregnancy, menopause and also associated with obesity, insulin resistance, and metabolic syndrome (1–3). On the other hand, pathological causes of goiter include genetic causes, inflammatory conditions, autoimmune disorders, and rarely thyroid hormone resistance and thyrotropin thyroid stimulating hormone (TSH)-secreting pituitary tumors (4, 5).

Depending on the pathophysiology, goiters can be diffuse, involving the whole gland or localized as in case of nodular goiter. The clinical presentation can vary based on secretion of the thyroid hormones (TH) from euthyroidism, hypothyroidism, to hyperthyroidism and with the size and location of the goiter. The most common type of goiter clinically encountered in the absence of inflammation and thyroid dysfunction is the simple nontoxic goiter that is classified as endemic and sporadic goiter. Although endemic goiter, due to iodine deficiency accounts for the higher prevalence worldwide, not all causes of goiter can be tracked back to iodine deficiency. A large number of cases with sporadic goiter are noted due to the presence of goitrogens and endocrine disruptors (5–7). In contrast, excessive doses of iodine have been shown to counteract the thyroid’s response to TSH and may even cause thyroid cell apoptosis (8).

The development of goiter involves global activation of thyroid epithelial cell proliferation following iodine deficiency or other goitrogenic stimuli. Benign goiter is an adaptive response of thyroid follicular cells to process that inhibit or diminishes TH production with a compensatory elevation of TSH to maintain normal circulating TH levels. This elevated TSH levels even though minimal causes significant increases in thyroid volume due to its proliferative and mitotic actions (5, 9). Long standing cases of goiter are known to result in developing thyroid nodules as a result of differential stimulation of the thyroid follicles. According to the Framingham study, new nodules emerged at a rate of 1 per 1000 each year, putting the lifetime risk of developing a nodule between 5 and 10% (10). People with central obesity have a 1.6 times higher risk of developing goter and thyroid nodules (11–13). In addition to the higher TSH, hyperinsulinemia found in individuals with obesity has been implicated with the higher incidences of thyroid nodules. Under these conditions, the mitogenic action of insulin and the increased availability of insulin-like growth factor-1 may contribute to the development of nodular hyperplasia (14). In a recent study, we had used the MALDI imaging technique to identify discriminatory peptides that were significantly altered in patients with goiter between lean and obese groups (15).

Proteomics has become one of the most important disciplines for characterizing cellular protein composition and providing insight into the mechanisms of biological processes in a high-throughput manner. Mass spectrometry proteomic techniques, using an untargeted approach provide an unbiased approach to comprehensively profile all the proteins within a cellular organelle, cell, or a tissue. These approaches can be used for proteome profiling, comparative expression analysis between the disease states (16, 17). Few studies have evaluated the changes in circulating proteome in thyroid pathology and have explored plasma proteome changes in patients with hypothyroidism and hyperthyroidism before and after treatment (18–20). On the other hand, a large body of literature has looked at the differences in the proteomic profiles of thyroid disease with a focus on benign nodules and malignancy (21–25) or normal thyroid tissue and malignant tissues (26–29). The current study aims to compare the tissue proteome of benign goiter and normal thyroid using a 2D-DIGE mass spectrometry proteomics approach. Finding the proteomic differences between the normal and the goitrogenous thyroid tissue will further our understanding of the changes occurring at the cellular and protein level in patients with goiter.

## Subjects and Methods

### Ethical approval and informed consent

The study procedures and protocols, including clinical samples, were reviewed and approved by the Institutional Review Board of the College of Medicine, King Saud University, Riyadh, Saudi Arabia (registration no. E-10-172). The patients/participants provided their written informed consent to participate in this study.

### Study design and patient selection

Thyroid tissue samples, from age and sex matched, patients with BDG (n=7) and controls (n=7) were obtained at the time of surgery from patients referred to the outpatient endocrine breast and surgery clinic at King Khalid University Hospital (KKUH) with complaints of benign goiter. Tissue samples were obtained from each patient with BDG at the time of total thyroidectomy. The control samples were collected from patients undergoing surgery for benign adenoma from the normal thyroid lobe by our collaborating surgeon. A 100 mg of tissue was excised from thyroid gland and was snap freezed in liquid nitrogen and stored until analysis. Venipuncture was used to obtain blood samples into plain tubes (Vacutainer, BD Biosciences, CA, USA) from each patient after a 10 hour fast for routine biochemical measurements and evaluation of the thyroid profile. Biochemical investigations were carried out using a Siemens Healthcare -Dimension Xpand Plus integrated autoanalyzer (Diagnostics, IL, USA). The sample size was calculated using a power analysis with the Progenesis SameSpots non-linear dynamics statistical software to determine the minimum number of biological replicates required.

### Tissue collection and protein extraction

Proteins extraction from thyroid tissue samples were carried out using T25 digital ULTRA TURRAX homogenizer (IKA, Germany) in lysis buffer (0.5 ml, pH 8.8, 30 mM Tris-HCl, 7 M urea, 2 M thiourea, 2% CHAPS, and a 1x protease inhibitor mix) on ice. The suspension was shaken at room temperature for 1 hour before being sonicated (Microsonicator, Qsonica Sonicators, USA; 30% pulse, two intervals of 1 min each, separated by a 1 min gap). Dithiothreitol (DTT, Fifty mM) was then added and the protein extracts centrifuged (4°C, 20,000 ×g, 40 mins). The impurities were removed, and the supernatants were cleaned using a 2D clean-up kit, following the manufacturer’s instructions (GE Healthcare, Sweden) (30, 31).

### Cye dye labelling, two-dimensional electrophoresis and image scanning

The protein pellets (each sample) were re-suspended in labelling buffer (7 M urea, 4% CHAPS, 2 M thiourea, 30 mM Tris) and a pH of 8.5 was adjusted. Protein concentrations were determined in triplicate using the 2D-Quantkit (GE Healthcare, Sweden). Labelling of proteins were performed using CyDye™ DIGE Fluor minimal dyes (400 pmol/50 μg, GE Healthcare, Sweden) as previously described by our group (20, 21). In summary, for each sample 50 ug of protein was incubated (on ice, in the dark, 30 mins) with 400 pmol of Cy-5/Cy3 freshly dissolved in anhydrous dimethyl formamide (DMF). The addition of lysine (1.0 L, 10 mM, 10 min, on ice, in the dark) terminated the process. Each sample was covalently labeled with either a Cy3 or a Cy5 flurophore. A pooled internal standard containing 50 ug of total protein from each sample was labeled with Cy2. As previously described (20, 21), the labeled samples were pooled according to the experimental design (**Table S1**) and ran on the same gel for comparison.

Analytical gel electrophoresis (First dimension) and Second dimension sodiumdodecyl sulfate polyacrylamide gel electrophoresis (SDS-PAGE) was performed as previously reported (20, 21). Sapphire Biomolecular Imager (Azure Bio systems, OH, USA) was used for scanning the gels analyzed using image analysis software Sapphire Capture system (Azure Biosystems,OH, USA). Total protein (1 mg) was extracted from a pool of equal amount of protein from the 14 thyroid tissue samples (7 BDG and 7 Control) to make preparative gels. Gels were stained for 5 days, then rinsed briefly with Milli-Q water and stored until the spots could be excised and identified by MS as described previously by our group (30, 31).

### Statistical analysis

An automatic spot detection method was employed to evaluate the gel images (2D-DIGE) using Progenesis SameSpots software (Nonlinear Dynamics, UK). Gel warping, DIGE normalization, and comparison modules are included in the package. To verify that no data was lost, all gel images were matched to a reference gel and superimposed. Log transformation of the spot volumes was performed to generate normally distributed data. Log normalized volume (LNV) was used to quantify differential expression. The BDG and control groups were directly compared, and one-way ANOVA was used to calculate fold difference values and p-values. Before applying the statistical criteria (ANOVA, p ≤ 0.05 and fold ≥1.5), all spots were pre-filtered and manually examined. In statistical processing, normalized spot volumes were used instead of spot intensities. Only those spots were submitted for MS analysis that met the following statistical criteria.

### Protein identification (MALDI-TOF MS)

Manually excised Coomassie stained gel spots were washed and digested according previously reported studies (20, 21). The mixture of tryptic peptides (0.8 μL) derived from each protein was spotted onto a MALDI target (384 MTP Anchorchip; 800 μm Anchorchip; Bruker Daltonics, Bremen, Germany). MALDI-MS(/MS). As previously mentioned, spectra were obtained using an UltraflexTerm time-of-flight (TOF) mass spectrometer equipped with a LIFT-MS/MS device (Bruker Daltonics) at 21 kV reflector and 17 kV detector voltages, respectively (20, 21). PMFs were calibrated against a standard (peptide calibration standard II, Bruker Daltonics). Flex Analysis software was used to assess the PMFs (Bruker Daltonics v.2.4). Interpretation of MS data was done using BioTools v3.2 (Bruker Daltonics). The peptide masses were searched against the Mascot search algorithm (v2.0.04, updated on 09/05/2021; Matrix Science Ltd., UK). Identified proteins were accepted as correct if they the Mascot score > 56. Because some proteins were in low abundance and did not give sufficiently powerful mass fingerprints, not all spots of interest could be recognized; some spots were mixtures of multiple proteins (30, 31).

### Bioinformatics analysis

Ingenuity pathway analysis (IPA) v9.0 (Ingenuity Systems, CA, USA) was employed to analyze interaction of protein networks and the functions of tissue proteins that were differentially expressed in BDG and control samples. UniProt IDs are mapped by IPA into the ingenuity knowledge base, which is the world’s largest manually curated resource containing data from all published scientific studies. This software also determines the functions and pathways which are significantly connected with the MS-generated protein list by matching the experimental expression data onto networks constructed from interactions published. Additionally, using the PANTHER (http://www.pantherdb.org) the detected proteins were grouped into distinct groups based on their molecular function and biological function.

## Results

### Clinical and biochemical data

The clinical and biochemical characteristics of age and BMI matched groups are shown in **Table 1**. None of our patients with benign diffuse goiter (BDG) showed any alteration in their thyroid profile and had no clinical evidence of increased thyroid antibodies. Histological findings from the post total thyroidectomy specimens revealed presence of large hyperplastic thyroid follicles with abundant colloid.

**Table 1:** Mean, SD and P values of biochemical characteristics of study subjects.

	Mean ± SDControls	Mean ± SDBDG	P values
N	7	7	
Age (years)	42.3 ± 12.2	37.3 ± 8.9	0.32
Gender (male/female)	5/2	4/3	
BMI	27.2 ± 1.5	29.5 ± 2.3	0.81
Urea (mmol/L)	4.7 ± 0.7	4.6 ± 0.9	0.81
Creatinine (mmol/L)	72.5 ± 13.1	76.1 ± 13.3	0.61
Aspartate transaminase (IU/L)	33.4 ± 6.6	35.7 ± 9.0	0.59
Fasting glucose (mmol/L)	5.3 ± 0.4	5.0 ± 0.5	0.23
Alkaline phosphatase (IU/L)	94.9 ± 25.9	96.8 ± 31.2	0.91
Alanine transaminase (IU/L)	18.0 ± 5.8	17.4 ± 3.9	0.81
TSH (mIU/l)	1.2 ± 2.1	1.9 ± 0.9	0.41
FT4 (pmol/L)	14.3 ± 5.5	15.8 ± 5.7	0.62
Triglycerides (mmol/L)	1.2 ± 0.3	1.4 ± 0.3	0.21
LDL cholesterol (mmol/L)	1.2 ± 0.4	1.0 ± 0.3	0.31
Total Cholesterol (mmol/L)	4.6 ± 0.6	4.8 ± 0.7	0.57
HDL cholesterol (mmol/L)	2.9 ± 0.8	3.0 ± 0.6	0.79

### Proteomic analysis of differentially expressed proteins (2D-DIGE)

**Figure 1:**
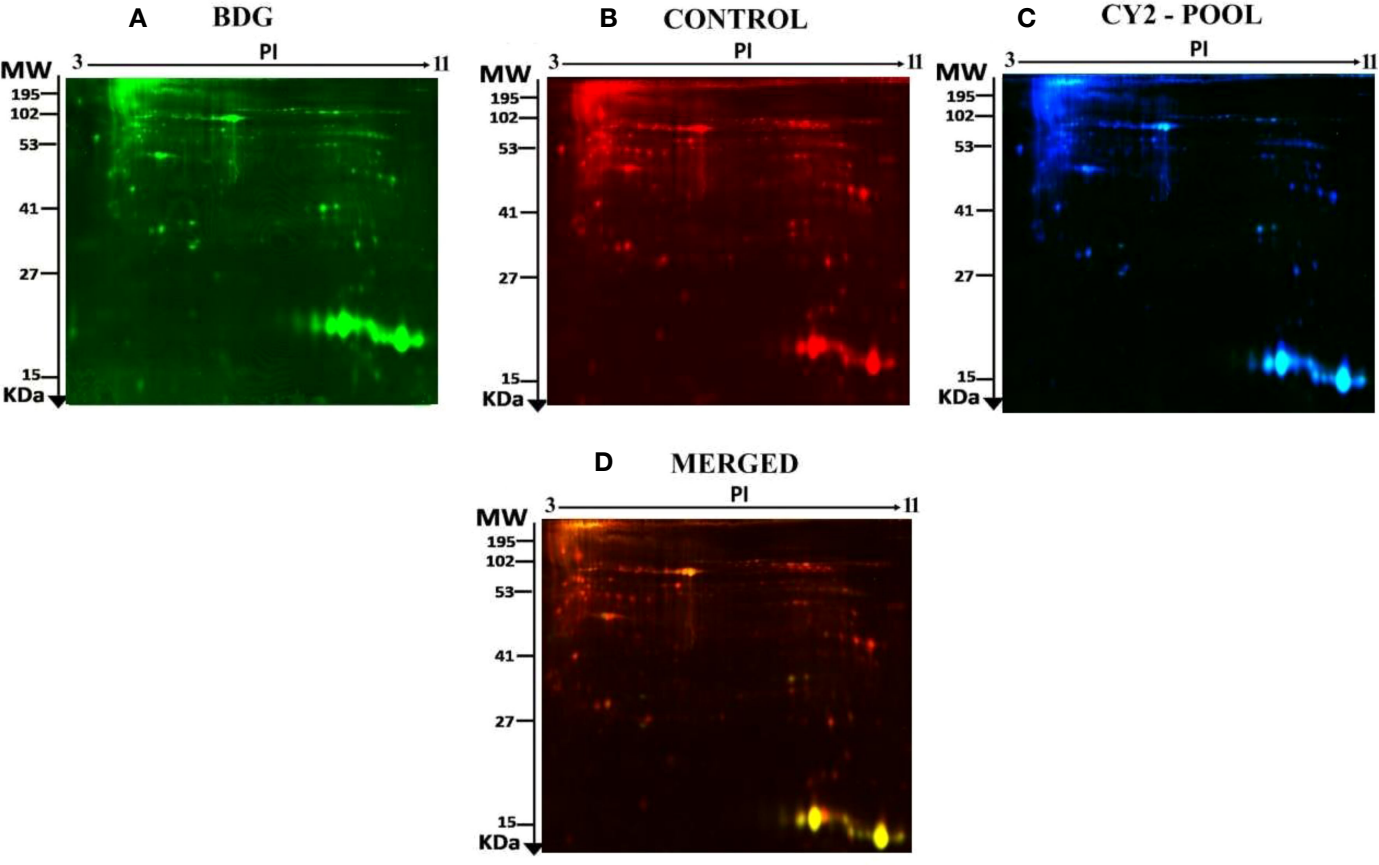
Representative fluorescent protein profile of a 2D-DIGE comprising tissue samples from BDG patients labelled with Cy3 **(A)**, Cy5 **(B)**, pooled internal control labelled with Cy2 **(C)**, and merged image **(D)**.

**Figure 2:**
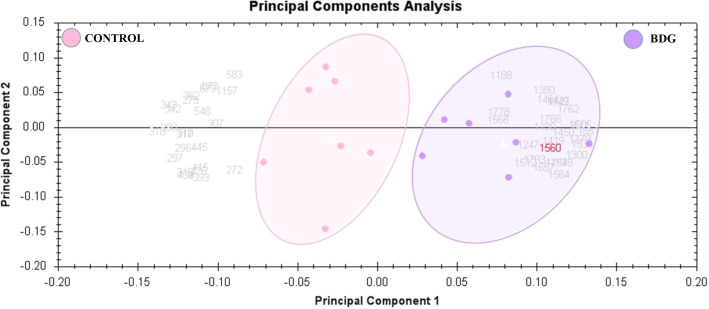
The two first principle components are plotted on a PCA. Together, they explained 82 percent of the variability in the chosen spot. Gels and spots are represented as colored dots and numerals, respectively.

To determine the proteins differentially expressed in BDG compared to control group, thyroid tissue samples from BDG (n = 7) and control (n = 7) were compared by 2D-DIGE. Progenesis Same Spots v3.3 software (Nonlinear Dynamics Ltd, UK) was used for analyses of gel images. Automated image analysis detected a total of 1150 spots on the gels among which 152 were statistically significant with ANOVA, P ≤ 0.05; fold-change ≥ 1.5 between the BDG and control samples. Representative fluorescent protein profiles of a 2D-DIGE of BDG samples labeled with Cy3 (**Figure 1A**), control samples labeled with Cy 5 (**Figure 1B**), pooled internal control labeled with Cy2 (**Figure 1C**), and representative overlay of Cy3/Cy5/Cy2 images are shown in **Figure 1**. (**Figure 1D**). The gels revealed a total of 152 spots that were significantly different (ANOVA, P ≤ 0.05; fold-change ≥ 1.5) between the BDG and control samples (**Figure 2**). The spot patterns were consistent across all 14 image gels, allowing them to be aligned and analyzed further. An internal standard with Cy2-labelling was used to achieve normalization across the entire gel sets and differential analysis (quantitative) of the protein levels. The 152 spots on the preparative gel with statistical significance between the BDG and controls were then excised for identification of proteins by MALDI-TOF MS.

Peptide mass fingerprinting (PMFs) recognized 90 of the 152 protein spots excised from preparative gel; MALDI-TOF mass spectrometry identified 61 spots as unique protein sequences, which Mascot matched with high confidence to entries in the SWISS-PROT database (**Table 2**, **Table S2**). The sequence coverage of the identified proteins ranged from 10% to 95%. Variants of the same protein were detected in many locations on the gel in a few cases (**Table 2**, **Figure S1**). Among the 90 proteins identified, 46 protein spots were up-regulated, 44 were downregulated in tissue samples of BDG patients compared to control. Among the significantly upregulated proteins included Fatty acid-binding protein, adipocyte (up 4.09 fold, p = 6.67E-04), Hormonally up-regulated neu tumor associated kinase (up 3.07 fold, p = 0.007), Actin, cytoplasmic 1 (up 2.92 fold, p = 0.02), Unconventional myosin-Ih (up 2.82 fold, p = 0.035), Actin, alpha cardiac muscle 1 (up 2.42 fold, p = 0.019), Peroxiredoxin-2 (up 2.16 fold, p = 0.007), Superoxide dismutase (Cu-Zn) (up 1.98 fold, p = 8.42E-04), Glyceraldehyde-3-phosphate dehydrogenase (up 1.59 fold, p = 0.046), (see **Table 2** for complete list). The significantly downregulated proteins included Thyroglobulin (down 2.34 fold, p = 0.004), Serine/threonine kinase greatwall (down 2.33 fold, p = 0.048), Actin, alpha skeletal muscle (down 2.11, p = 0.02), Protein disulfide-isomerase A32 (down 2.06 fold, p = 0.01), Fructose-bisphosphate aldolase A (down 2.04 fold, p = 0.02) and Vimentin (down 1.78 fold, p = 0.04) (**Table 2**, **Table S2**). Among the identified proteins, Fibrinogen beta chain, ATP synthase subunit alpha, mitochondrial, Thyroglobulin, Peroxiredoxin-2, Immunoglobulin heavy constant gamma 1, Unconventional myosin-Ic, Actin, Actin, Alpha skeletal muscle, Alpha cardiac muscle 1, DNA repair protein XRCC3, Peroxiredoxin-1, Parkinson disease 7, Ras-related protein Rab-14, Alpha-enolase, Hemoglobin subunit beta and Methanethiol oxidase were found in more than one spot on the gels, which could be associated with the cleavage by enzymes,post-translational modifications, or the presence of different protein species.

**Table 2:** Proteins identified in thyroid tissue samples with differences in abundance between BDG and control states.

Sl no:	Spot No^a^	Accession No	Protein Name	MASCOT ID	P value^b^(ANOVA)	Ratio BDG/C^c^	Exp^d^
1	1560	P32119	Peroxiredoxin-2	PRDX2_HUMAN	5.35E-04	1.76	UP
2	1651	P15090	Fatty acid-binding protein, adipocyte	FABP4_HUMAN	6.67E-04	4.09	UP
3	1605	P00441	Superoxide dismutase (Cu-Zn)	SODC_HUMAN	8.42E-04	1.98	UP
4	343	P02675	Fibrinogen beta chain	FIBB_HUMAN	0.002	-2.05	DOWN
5	449	P10809	60 kDa heat shock protein, mitochondrial	CH60_HUMAN	0.002	-1.94	DOWN
6	1300	Q07890	Son of sevenless homolog 2	SOS2_HUMAN	0.002	2.37	UP
7	318	P01859	Immunoglobulin heavy constant gamma 2	IGHG2_HUMAN	0.002	-1.8	DOWN
8	1157	P04075	Fructose-bisphosphate aldolase A	ALDOA_HUMAN	0.002	-2.04	DOWN
9	1657	Q3KQZ1	Solute carrier family 25 member 35	S2535_HUMAN	0.003	2.06	UP
10	317	P25705	ATP synthase subunit alpha, mitochondrial	ATPA_HUMAN	0.003	-1.94	DOWN
11	1427	O95833	Chloride intracellular protein 3	CLIC3_HUMAN	0.003	1.9	UP
12	342	Q16851	UTP—glucose-1-phosphate uridylyltranferase	UGPA_HUMAN	0.003	-1.82	DOWN
13	548	P01266	Thyroglobulin	THYG_HUMAN	0.004	-2.34	DOWN
14	1419	P02647	Apolipoprotein A-I	APOA1_HUMAN	0.006	1.77	UP
15	1380	Q13162	Peroxiredoxin-4	PRDX4_HUMAN	0.006	2.19	UP
16	297	P25705	ATP synthase subunit alpha, mitochondrial	ATPA_HUMAN	0.006	-1.72	DOWN
17	1778	P20472	Parvalbumin alpha	PRVA_HUMAN	0.006	1.84	UP
18	1548	P32119	Peroxiredoxin-2	PRDX2_HUMAN	0.007	2.16	UP
19	1564	P57058	Hormonally up-regulated neu tumor associated kinase	HUNK_HUMAN	0.007	3.07	UP
20	1568	P32119	Peroxiredoxin-2	PRDX2_HUMAN	0.007	1.67	UP
21	404	P30101	Protein disulfide-isomerase A3	PDIA3_HUMAN	0.008	-2.31	DOWN
22	296	P01857	Immunoglobulin heavy constant gamma 1	IGHG1_HUMAN	0.008	-1.68	DOWN
23	403	Q14145	Kelch-like ECH-associated protein 1	KEAP1_HUMAN	0.008	1.84	UP
24	1511	Q8TED0	U3 small nucleolar RNA-associated preotein 15 homolog	UTP15_HUMAN	0.009	2.02	UP
25	362	P01857	Immunoglobulin heavy constant gamma 1	IGHG1_HUMAN	0.01	-1.62	DOWN
26	393	P30101	Protein disulfide-isomerase A3	PDIA3_HUMAN	0.011	-2.06	DOWN
27	316	P01857	Immunoglobulin heavy constant gamma 1	IGHGI_HUMAN	0.012	-2.03	DOWN
28	310	Q6RFH5	WD repeat-containing protein 74	WDR74_HUMAN	0.013	-1.82	DOWN
29	1451	P04792	Heat shocked protein beta-1	HSPB1_HUMAN	0.014	1.94	UP
30	673	P00738	Heptoglobin	HPT_HUMAN	0.014	-1.6	DOWN
31	275	P01857	Immunoglobulin heavy constant gamma 1	IGHG1_HUMAN	0.015	-1.97	DOWN
32	272	P02768	Albumin	ALBU_HUMAN	0.015	-2.3	DOWN
33	415	O00159	Unconventional myosin-Ic	MYO1C_HUMAN	0.015	1.91	UP
34	1188	P63244	Receptor of activated protein C kinase 1	GBLP_HUMAN	0.016	1.8	UP
35	476	Q9Y646	Carboxypeptidase Q	PGCP_HUMAN	0.017	-1.66	DOWN
36	1512	P07741	Adenine phosphoribosyltransferase	APT_HUMAN	0.019	1.98	UP
37	1247	P22626	Heterogeneous nuclear ribonucleoprotein A2/B1	ROA2_HUMAN	0.019	2.42	UP
38	1650	Q6p5S2	Protein LEG1 homolog	LEG1_HUMAN	0.019	1.5	UP
39	1609	P68032	Actin, alpha cardiac muscle 1	ACTC _HUMAN	0.019	2.42	UP
40	573	P68133	Actin, alpha skeletal muscle	ACTS_HUMAN	0.02	-2.11	DOWN
41	366	P30101	Protein disulfide-isomerase A3	PDIA3_HUMAN	0.021	-1.65	DOWN
42	406	O43542	DNA repair protein XRCC3	XRCC3_HUMAN	0.021	-1.76	DOWN
43	1496	Q06830	Peroxiredoxin-1	PRDX1_HUMAN	0.021	2.01	UP
44	1553	Q99497	Parkinson disease 7	PARK7_HUMAN	0.022	1.61	UP
45	1491	P61106	Ras-related protein Rab-14	RAB14_HUMAN	0.024	2.02	UP
46	1609	P68032	Actin, alpha cardiac muscle 1	ACTC _HUMAN	0.019	2.42	UP
47	573	P68133	Actin, alpha skeletal muscle	ACTS_HUMAN	0.02	-2.11	DOWN
48	366	P30101	Protein disulfide-isomerase A3	PDIA3_HUMAN	0.021	-1.65	DOWN
49	406	O43542	DNA repair protein XRCC3	XRCC3_HUMAN	0.021	-1.76	DOWN
50	1496	Q06830	Peroxiredoxin-1	PRDX1_HUMAN	0.021	2.01	UP
51	1553	Q99497	Parkinson disease 7	PARK7_HUMAN	0.022	1.61	UP
52	1491	P61106	Ras-related protein Rab-14	RAB14_HUMAN	0.024	2.02	UP
53	1615	Q01469	Fatty acid-binding 5	FABP5_HUMAN	0.019	2.16	UP
54	1289	P60709	Actin, cytoplasmic 1	ACTB_HUMAN	0.02	2.92	UP
55	412	P30101	Protein disulfide-isomerase A3	PDIA3_HUMAN	0.021	-1.63	DOWN
56	1594	Q99497	Parkinson disease 7	PARK7_HUMAN	0.021	2.08	UP
57	1586	Q99497	Parkinson disease 7	PARK7_HUMAN	0.021	1.51	UP
58	291	Q9UHG3	Prenylcysteine oxidase 1	PCYOX_HUMAN	0.022	-1.85	DOWN
59	397	P30101	Protein disulfide-isomerase A3	PDIA3_HUMAN	0.022	-1.63	DOWN
60	1699	Q8N1T3	Unconventional myosin-Ih	MYO1H_HUMAN	0.035	2.82	UP
61	631	P19652	Alpha-1-acid glycoprotein 2	A1AG2_HUMAN	0.036	1.61	UP
62	597	P06733	Alpha-enolase	ENOA_HUMAN	0.036	1.64	UP
63	1634	P68871	Hemoglobin subunit beta	HBB_HUMAN	0.036	1.86	UP
64	340	Q6PCB0	Von willebrand factor A domain containing protein 1	VWA1_HUMAN	0.036	-1.71	DOWN
65	764	P33176	Kinesin-1 heavy chain	KINH_HUMAN	0.037	1.62	UP
66	1637	P68871	Hemoglobin subunit beta	HBB_HUMAN	0.037	1.77	UP
67	334	P68871	Hemoglobin subunit beta	HBB_HUMAN	0.039	-1.99	DOWN
68	421	P30101	Protein disulfide-isomerase A3	PDIA3_HUMAN	0.039	-1.63	DOWN
69	1664	Q96N38	Zinc finger protein	ZN714_HUMAN	0.039	1.5	UP
70	324	P10109	Adrenodoxin, mitochondrial	ADX_HUMAN	0.039	-1.79	DOWN
71	564	P08670	Vimentin	VIME_HUMAN	0.04	-1.78	DOWN
72	345	Q8NB42	Zinc finger protein 527	ZN527_HUMAN	0.04	-1.69	DOWN
73	315	P02675	Fibrinogen beta chain	FIBB_HUMAN	0.041	-1.77	DOWN
74	344	O00159	Unconventional myosin-Ic	MYO1C_HUMAN	0.041	1.68	UP
75	292	Q07507	Dermatopotin	DERM_HUMAN	0.042	-1.93	DOWN
76	972	P06733	Alpha-enolase	ENOA_HUMAN	0.043	1.53	UP
77	460	P01266	Thyroglobulin	THYG_HUMAN	0.044	-2.08	DOWN
78	699	P01266	Thyroglobulin	THYG_HUMAN	0.044	-1.62	DOWN
79	409	Q66GS9	Centrosomal protein of 135 kDa	CP135_HUMAN	0.044	-1.92	DOWN
80	247	A8MUN3	Putative uncharacterized protein ENSP00000381830	YQ048_HUMAN	0.045	-1.8	DOWN
81	1627	P15090	Fatty acid-binding protein, adipocyte	FABP4_HUMAN	0.046	1.75	UP
82	1259	P04406	Glyceraldehyde-3-phosphate dehydrogenase	G3P_HUMAN	0.046	1.59	UP
83	`308	Q13228	Methanethiol oxidase	SBP1_HUMAN	0.047	-1.73	DOWN
84	1603	P30626	Sorcin	SORCN_HUMAN	0.048	2.24	UP
85	1465	Q1A5X7	Putative WASP homolog-associated protein with actin, membranes and microtubules-like protein 1	WHAL1_HUMAN	0.048	1.69	UP
86	328	Q96GX5	Serine/threonine kinase greatwall	MASTL_HUMAN	0.048	-2.33	DOWN
87	1426	P02743	Serum amyloid P-component	SAMP_HUMAN	0.049	1.5	UP
88	969	P14550	Aldo-keto reductase family 1 member A1	AK1A1_HUMAN	0.05	-1.57	DOWN
89	359	Q13228	Methanethiol oxidase	SBP1_HUMAN	0.05	-1.73	DOWN
90	983	P40121	Macrophage-capping protein	CAPG_HUMAN	0.050	-1.79	DOWN

a Protein accession number.

b P-Value (ANOVA).

c Ratio BDG/control group.

d Protein expression between BDG and control group. Using 2D-DIGE-MALDI TOF, average ratio values between the two states are displayed together with their associated fold changes and one-way ANOVA (fold-change ≥ 1.5, P ≤ 0.05). Database: SwissProt; taxonomy: Homo sapiens.

### Principal component and cluster analysis

**Figure 3:**
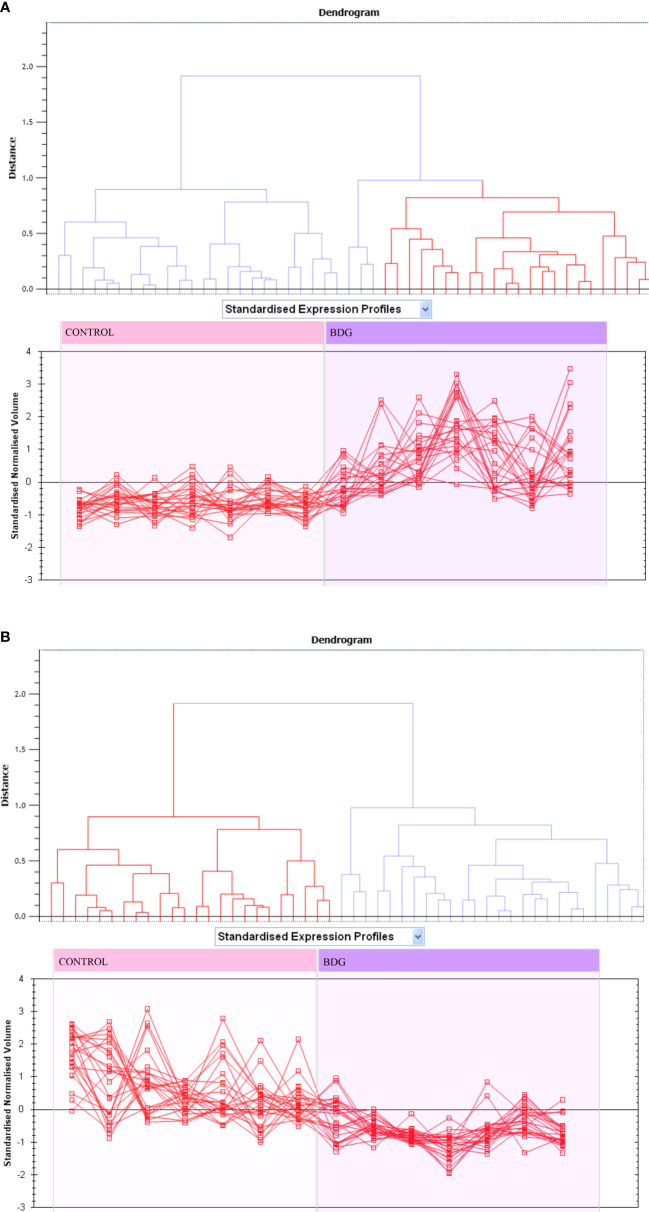
Expression profiles divided into expression pattern clusters, with the number of spots for each cluster indicated. Each line indicates a spot’s standardized abundance covering all gels and corresponds to one among the clusters created by hierarchical cluster analysis. The spots with increased abundance indicate the 46 proteins up regulated **(A)**. The spots with decreased abundance indicate the 44 proteins downregualted in thyroid tissue from patients with BDG compared to control states **(B)** (Progenesis SameSpots).

**Figure 4:**
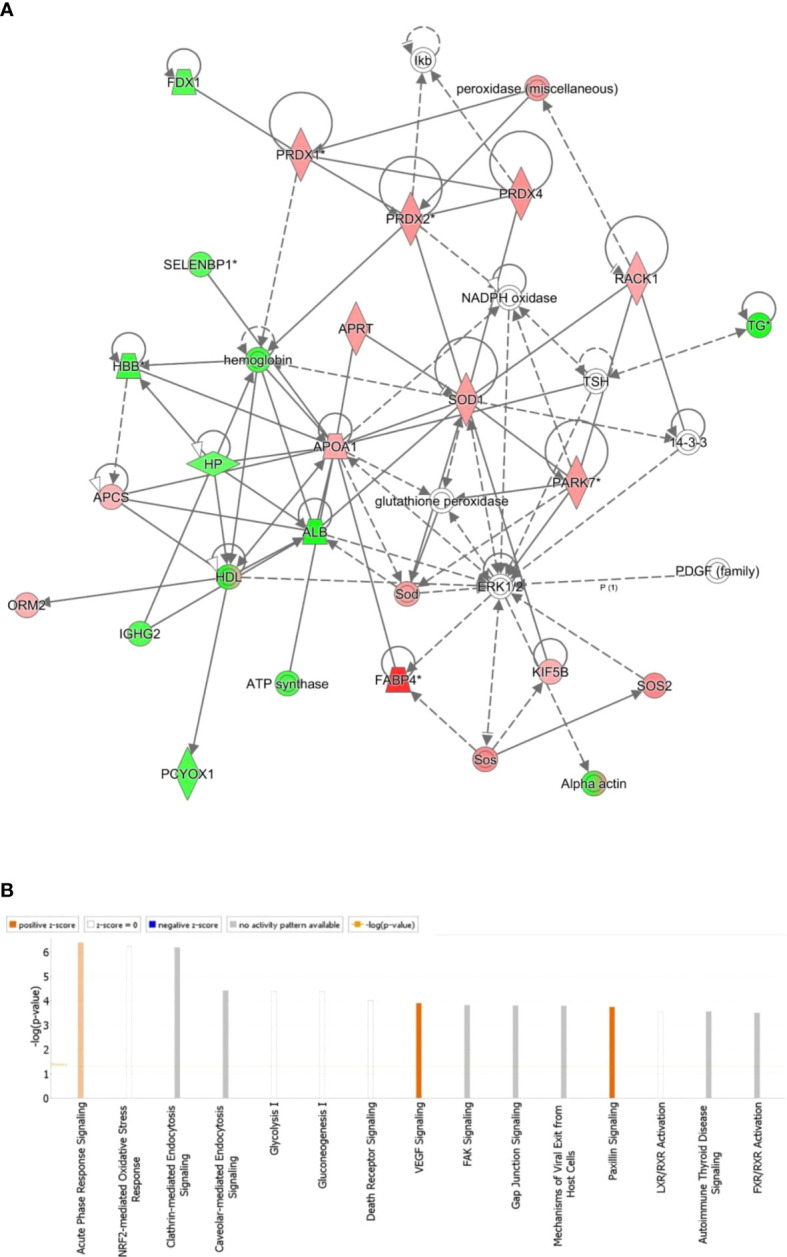
The most enriched interaction network of the differentially expressed proteins in BDG compared to the control thyroid states. Red nodes indicate up-regulated; green nodes indicate down-regulated. The central nodes in th the pathway related to signaling of the ERK1/2, Glutathione peroxidase and NADPH oxidase were found to be deregulated between the two groups. Uncolored nodes indicate potential targets that were functionally matched with the differentially expressed proteins. Solid lines indicates direct molecular connections, whereas dashed lines denotes indirect interactions **(A)**. The figure depicts the top 15 canonical pathways as graded by the IPA’s P-values **(B)**.

Principal component analysis (PCA) performed on all 90 spot features demonstrated significant (p < 0.05 by ANOVA) changes in abundance, as identified by MS. Also, Furthermore, PCA demonstrated that the two groups clustered significantly differently based on distinct proteins, with an 82 percent cutoff score (**Figure 3**). Based on hierarchical clustering analysis, differentially abundant spots showed clusters of expression patterns (**Figures 4A, B**). The clustering pattern revealed that the change in protein intensity between BDG and control samples was significantly different for selected spots.

### Protein interaction analysis

All 90 differentially regulated proteins were submitted to bioinformatic analysis using IPA. The analysis revealed that 29 proteins interacted directly/indirectly via protein networks (**Figure 4A**). To construct a protein-protein interactions network, the software computes a score based on the best fit from the input data set and the biological functions database. The resulting network is enriched for proteins with interactions, where the interacting proteins are represented by nodes and a line denotes their biological relationships. Fifteen interaction networks were identified for the proteins exhibiting differential expression profile. The proposed highest interaction network pathway (Score=48) was related to Organismal Injury and Abnormalities, Endocrine System Disorders and Cancer. Only the most important paths are displayed (**Figure 4A**). **Figure 4B** shows the canonical pathways that are enriched in the current dataset. In **Figure 4B**, the canonical pathways are sorted by decreasing log (p-value) of enrichment. Acute Phase Response Signaling (p value: 4.08E-07, overlap: 3.9%), NRF2-mediated Oxidative Stress Response (p value: 5.68E-07, overlap: 3.7%), and Clathrin-mediated Endocytosis Signaling (p value: 6.54E-07, overlap: 3.6 percent) were the three most interesting enriched canonical pathways. Canonical pathways identified in the study are summarized in (**Figure S2**).

### Functional classification of key proteins

**Figure 5:**
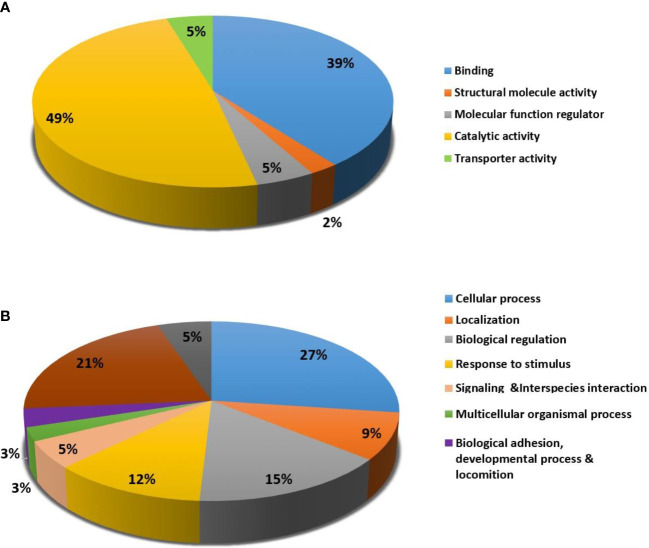
Comparative representation (percentage) of identified proteins divided into groups based on their Molecular function **(A)** and Biological process **(B)**.

For the classification of identified proteins according to their molecular function (**Figure 5A**) and biological function, the Protein Analysis Through Evolutionary Relationships (PANTHER) system was utilized (**Figure 5B**). The majority of the differentially expressed proteins were enzymes with catalytic activity (49%) and binding proteins (39%) according to the functional category (**Figure 5A**). In terms of biological activities, the majority of the proteins found were engaged in cellular processes (27%) and localisation (21%) respectively (**Figure 5B**).

## Discussion

The application of proteomic analysis yielded protein fingerprints displaying a pattern of differential expression of proteins between the BDG compared to the normal thyroid tissue. A diffuse nontoxic goiter is defined as a diffuse enlargement of the thyroid without nodules or hyperthyroidism. Because there are no nodules, this is referred to as ‘simple goiter,’ or ‘colloid goiter,’ because uniform follicles filled with colloid are present (32). The significantly different protein expressions found in the BDG tissues reflect the underlying molecular features of its probable pathology. Our results indicate that BDG tissues are characterized by an increase in the proteins involved in increasing the redox state of the thyrocytes, and chaperone proteins involved in regulating protein folding and glycosylation. It is also reflected in the pathway analysis. The central nodes with the highest connectivity in the IPA analysis were linked around activity of NADPH oxidase, glutathione peroxidase and ERK1/2 signaling pathways. Dysregulation of metabolic pathways related to glycolysis, and the proteins involved in maintenance of the cytoskeletal structure of the thyrocytes were also identified.

### Proteins involved in regulating oxidative stress

The proteomic analysis of BDG thyroid tissues compared to the controls showed an increase in abundance of enzymes regulating the redox state of the thyrocytes. These included peroxiredoxin-1,2, and 4, superoxide dismutase (SOD), PARK 7, SOS and RAB14. TH synthesis requires the presence of the reactive oxygen species (ROS) radicals, especially hydrogen peroxide (H2O2) for oxidation of iodide, iodination of tyrosine residues and coupling of iodotyrosines to thyroglobulin (Tg) catalyzed by thyroid peroxidase (33, 34). H2O2 generation in the thyroid gland is usually carried out by the NADPH oxidases (NOX) and by superoxide dismutase. An increase in abundance of superoxide dismutase (Cu-Zn), also known as superoxide dismutase 1 (SOD1), catalyzes the conversion of superoxide to H2O2 was noted in our study (34). The increase in this enzyme could be an attempt by the thyrocytes to increase the TH synthesis in the patients with BDG. Additionally, two other proteins increased in our data set, Son of sevenless homolog 2 (SOS2) and RAB14 are known to interact with the NOX system and increase intracellular H2O2 and ROS levels intracellular. SOS2 is a guanine nucleotide exchange factor involved in the cell signalling, that activates the Ras-GTPases enzymes that directly stimulate the NOX enzymes (35, 36). The mechanism by which Ras controls the amounts of reactive oxygen species (ROS) is still unknown.

### Proteins involved in glycolysis and cytoskeletal proteins

The proteomic profiling of the BDG showed differential regulation in the levels of enzymes involved glycolysis compared to the controls. We noted an increase in the abundance of alpha enolase and glyceraldehyde 3 phosphate dehydrogenase (GAPDH) while levels of fructose bisphosphate aldolase were reduced. Previous studies have shown the involvement of these proteins in numerous diverse roles besides glycolysis. GAPDH in particular is known to facilitate a metabolic shift from anaerobic respiration to the pentose phosphate pathway that regenerates NADPH and aids in the maintenance of oxidation/reduction balance (37). Moreover, GAPDH and alpha enolase independent from the glycolytic function are also known to interact with the cytoskeletal proteins. Previous studies have demonstrated their binding with actin filaments facilitating microtubule bundling and actin polymerization, which in turn improves vesicular trafficking between cellular compartments (38, 39). In relation to this we also noted an increase in abundance of the actin proteins in our data set along with myosin. Both the cytoskeletal proteins are known to participate in the vesicular mediated transport and endocytosis (40, 41). Transport and release of TH as well processing of Tg molecules within the thyrocyte are dependent on vesicular transport. The increase in these proteins could be a probable mechanism aimed at increasing the TH synthesis and release by the BDG thyroid tissue.

### Proteins involved in regulation of thyroglobulin

We found a decrease in the abundance of Tg, protein disulfide-isomerase A3 and UTP-glucose-1 phosphate uridyl transferase (UGPA) in the BDG tissues. Tg is a large glycoprotein synthesized by the thyrocytes that serves as a matrix for thyroid hormones synthesis and storage in the follicles. Defects in Tg have been attributed to development of thyroid disorders including goitre and congenital hypothyroidism. Thyrocytes are responsible for the biosynthesis of Tg, which accounts for more than half of the thyroid gland’s total protein. Mature Tg is a heavily glycosylated molecule that is stored as colloid in the thyroid follicles. Defective glycosylation of the Tg prevents its effective iodination resulting in impaired TH synthesis. Glycan attachment is a multistep enzymatic process that includes the transfer of monosaccharides from activated nucleoside triphosphate donors to a sugar acceptor (42). A decrease in abundance of the glycosyltransferase enzyme, UGPA involved in catalyzing the key regulatory role in glycosylation was noted in our study (42). In addition to this enzyme our proteomic profile in BDG showed a decrease in another protein involved in regulation of Tg, namely protein disulfide-isomerase A3 (PDIA3). PDIA3 is a thiol oxidoreductase enzyme that functions as a chaperone protein which is involved in regulating the proper folding of thyroglobulin (43). Defects in the regulation of Tg synthesis result in defective iodination and coupling of the iodinated residues could account for the hypertrophy and hyperplasia of the thyroid.

### Other proteins

Besides the above mentioned protein we also found an increase in alpha-1-acid glycoprotein 2, an acute phase protein synthesized mainly by the hepatocytes. In-vitro studies have shown that alpha-1-acid glycoprotein 2 in low concentrations stimulated the effect of TSH and in high concentrations inhibited the effect of TSH (44). Serum amyloid P-component, a protein from the pentraxin family of proteins, was found expressed highly in the BDG tissues. Although there is no known function of human serum amyloid P-component protein, it is believed to contribute to innate immunity. It is also the main component of amyloid deposits in the cells (45). It is not clear whether the increased abundance of serum amyloid P-component protein in the nodules indicates amyloid deposition. Serine/threonine kinase greatwall, also known as Greatwall (GWL), is a regulator of mitosis. It is reported to be up-regulated in many cancer types and is found to be associated with aggressive malignancies (46). Our results show that GWL protein is downregulated in the BDG tissues. Apolipoprotein A-I is another protein that is found highly expressed in the nodules compared to normal thyroid tissue. It is a major component of high density lipoprotein particles. A higher abundance of Apolipoprotein A-I was reported previously in cold thyroid nodules (47). Another lipid transporting molecule, adipocyte fatty acid-binding protein, is also found to be high in the BDG tissues. Increased expression of lipid transporters in the nodules could be an indication of increased cholesterol uptake. Adipocyte fatty acid-binding protein is usually expressed in the adipocytes and macrophages. Ectopic expression of adipocyte fatty acid-binding protein in normal thyroid tissue has not been reported before. Expression of adipocyte fatty acid-binding protein in other tissues was found to be associated with tumor growth (48).

The present study has found that the oxidative systems which are required for thyroxine production are in an activated state in the BDG tissues in response to lower thyroglobulin production. In addition, the present study has unraveled some novel molecular features of the BDG tissues.

## Conclusion

The proteomics of BDG tissues reveals an increase in enzymes involved in regulating the oxidative balance in the thyrocytes. The increase in oxidative stress can be a compensatory mechanism to overcome the decreased and defective Tg synthesis in these cases.

## Data availability statement

The original contributions presented in the study are included in the article/**supplementary material**. Further inquiries can be directed to the corresponding author.

## Ethics statement

All procedures and protocols, including clinical samples, were reviewed and approved by the Institutional Review Board of the College of Medicine, King Saud University, Riyadh, Saudi Arabia (registration no. E-10-172). The patients/participants provided their written informed consent to participate in this study.

## Author contributions

AAA, HB and AM conceived the idea and designed the study. AAA, AM and AA were involved in patient recruitment. HB and MM performed the proteomics lab work. HB, AM, MM and AAA carried out data analysis. AM, HB, MR, MM and AAA wrote the manuscript. All authors have read and approved the final manuscript.

## Funding

This project was funded by the National Plan for Science, Technology and Innovation (MAARIFAH), King Abdulaziz City for Science and Technology, Saudi Arabia, (Project No. 08-MED513-02).

## Acknowledgments

We wish to acknowledge Mr. Majed Kenneth and Ms. Amina Fallata for their assistance in the laboratory work.

## Conflict of interest

The authors declare that the research was conducted in the absence of any commercial or financial relationships that could be considered as a potential conflict of interest.

## Publisher’s note

All claims expressed in this article are solely those of the authors and do not necessarily represent those of their affiliated organizations, or those of the publisher, the editors and the reviewers. Any product that may be evaluated in this article, or claim that may be made by its manufacturer, is not guaranteed or endorsed by the publisher.
