# Supplementary material for: Proteomic profiling of thyroid tissue in patients with obesity and benign diffuse goiter
Source: Front Endocrinol (Lausanne). 2022 Jul 28;13:923465. doi: 10.3389/fendo.2022.923465 (PMC9365950; doi:10.3389/fendo.2022.923465)
Supplement: Supplementary file 1 [file DataSheet_1.pdf]

SUPPLEMENTARY DATA:

Table S1: Dye-switching strategy applied during labeling to avoid dye-specific bias. A total of 14 patient samples (7 BDG and 7 control) were run on 7 2D-PAGE gels. Samples were labeled randomly with Cy3 and Cy5, and a pooled sample was used as an internal standard and was stained with Cy2 [BDG Benign diffuse goiter, C: Control]

Gel	Cy3	Cy5	Cy2
1	BDG1	C1	Pooled sample
2	C2	BDG2	Pooled sample
3	BDG3	C3	Pooled sample
4	C4	BDG4	Pooled sample
5	BDG5	C5	Pooled sample
6	C6	BDG6	Pooled sample
7	BDG7	C7	Pooled sample

Table S2: Mass spectrometry list of significant differentially abundant proteins between BDG and control states identified in thyroid tissue samples, using 2D-DIGE with. Protein name, accession number, Mascot score, MS % coverage, protein MW and pI values according to Uniprot database are listed.

NO.	Spot No[a]	Accession No[b]	MASCOT ID	Protein name	Pi[c]	MW[d]	Cov%	Score[e]
1	1560	P32119	PRDX2_HUMAN	Peroxiredoxin-2	5.66	22099	63	98
2	1651	P15090	FABP4_HUMAN	Fatty acid-binding protein, adipocyte	6.59	14824	31	57
3	1605	P00441	SODC_HUMAN	Superoxide dismutase (Cu-Zn)	5.70	16154	66	92
4	343	P02675	FIBB_HUMAN	Fibrinogen beta chain	8.54	56577	57	164
5	449	P10809	CH60_HUMAN	60 kDa heat shock protein, mitochondrial	5.70	61167	43	105
6	1300	Q07890	SOS2_HUMAN	Son of sevenless homolog 2	6.39	154251	15	57
7	318	P01859	IGHG2_HUMAN	Immunoglobulin heavy constant gamma 2	7.66	36505	33	58

8	1157	P04075	ALDOA_HUMAN	Fructose-bisphosphate aldolase A	8.30	39851	71	192
9	1657	Q3KQZ1	S2535_HUMAN	Solute carrier family 25 member 35	5.13	59020	21	60
10	317	P25705	ATPA_HUMAN	ATP synthase subunit alpha, mitochondrial	9.21	32641	39	59
11	1427	O95833	CLIC3_HUMAN	Chloride intracellular protein 3	9.16	59828	23	74
12	342	Q16851	UGPA_HUMAN	UTP—glucose-1-phosphate uridylyltranferase	5.99	26917	72	130
13	548	P01266	THYG_HUMAN	Thyroglobulin	8.16	57076	31	59
14	1419	P02647	APOA1_HUMAN	Apolipoprotein A-I	5.40	311608	15	70
15	1380	Q13162	PRDX4_HUMAN	Peroxiredoxin-4	5.56	30759	85	198
16	297	P25705	ATPA_HUMAN	ATP synthase subunit alpha, mitochondrial	5.86	30749	68	110
17	1778	P20472	PRVA_HUMAN	Parvalbumin alpha	9.16	59828	45	126
18	1548	P32119	PRDX2_HUMAN	Peroxiredoxin-2	4.98	12051	53	58
19	1564	P57058	HUNK_HUMAN	Hormonally up-regulated neu tumor associated kinase	5.66	22044	45	82
20	1568	P32119	PRDX2_HUMAN	Peroxiredoxin-2	9.24	80376	16	57
21	404	P30101	PDIA3_HUMAN	Protein disulfide-isomerase A3	5.66	22049	66	152
22	296	P01857	IGHG1_HUMAN	Immunoglobulin heavy constant gamma 1	5.98	57146	63	189

23	403	Q14145	KEAP1_HUMAN	Kelch-like ECH-associated protein 1	8.46	36596	46	76
24	1511	Q8TED0	UTP15_HUMAN	U3 small nucleolar RNA-associated preotein 15 homolog	6.00	71160	40	89
25	362	P01857	IGHG1_HUMAN	Immunoglobulin heavy constant gamma 1	9.18	58661	36	63
26	393	P30101	PDIA3_HUMAN	Protein disulfide-isomerase A3	8.46	57146	41	78
27	316	P01857	IGHGI_HUMAN	Immunoglobulin heavy constant gamma 1	5.98	57146	65	172
28	310	Q6RFH5	WDR74_HUMAN	WD repeat-containing protein 74			40	67
29	1451	P04792	HSPB1_HUMAN	Heat shocked protein beta-1	8.64	47985	22	62
30	673	P00738	HPT_HUMAN	Heptoglobin	5.98	22826	59	142
31	275	P01857	IGHG1_HUMAN	Immunoglobulin heavy constant gamma 1	6.13	45861	35	62
32	272	P02768	ALBU_HUMAN	Albumin	8.46	36596	39	66
33	415	O00159	MYO1C_HUMAN	Unconventional myosin-Ic	5.92	71317	60	187
34	1188	P63244	GBLP_HUMAN	Receptor of activated protein C kinase 1	9.48	22503	25	64
35	476	Q9Y646	PGCP_HUMAN	Carboxypeptidase Q	7.60	35511	68	105
36	1512	P07741	APT_HUMAN	Adenine phosphoribosyltransferase	5.79	52083	23	64
37	1247	P22626	ROA2_HUMAN	Heterogeneous nuclear ribonucleoprotein A2/B1	5.78	19766	79	108
38	1650	Q6p5S2	LEG1_HUMAN	Protein LEG1 homolog	8.97	37464	50	101

39	1609	P68032	ACTC _HUMAN	Actin, alpha cardiac muscle 1	5.34	15048	82	114
40	573	P68133	ACTS_HUMAN	Actin, alpha skeletal muscle	5.23	42334	40	84
41	366	P30101	PDIA3_HUMAN	Protein disulfide-isomerase A3	5.23	42366	47	146
42	406	O43542	XRCC3_HUMAN	DNA repair protein XRCC3	5.98	57146	68	210
43	1496	Q06830	PRDX1_HUMAN	Peroxiredoxin-1	8.81	38282	26	57
44	1553	Q99497	PARK7_HUMAN	Parkinson disease 7	8.27	22324	37	64
45	1491	P61106	RAB14_HUMAN	Ras-related protein Rab-14	6.33	20050	87	129
46	1609	P68032	ACTC _HUMAN	Actin, alpha cardiac muscle 1	5.23	42334	40	84
47	573	P68133	ACTS_HUMAN	Actin, alpha skeletal muscle	5.23	42366	47	146
48	366	P30101	PDIA3_HUMAN	Protein disulfide-isomerase A3	5.98	57146	68	210
49	406	O43542	XRCC3_HUMAN	DNA repair protein XRCC3	8.81	38282	26	58
50	1496	Q06830	PRDX1_HUMAN	Peroxiredoxin-1	8.27	22324	37	64
51	1553	Q99497	PARK7_HUMAN	Parkinson disease 7	6.33	20050	87	129
52	1491	P61106	RAB14_HUMAN	Ras-related protein Rab-14	5.85	24110	44	57
53	1615	Q01469	FABP5_HUMAN	Fatty acid-binding 5	6.60	15497	46	68
54	1289	P60709	ACTB_HUMAN	Actin, cytoplasmic 1	5.29	42052	58	132
55	412	P30101	PDIA3_HUMAN	Protein disulfide-isomerase A3	5.98	57146	61	167

56	1594	Q99497	PARK7_HUMAN	Parkinson disease 7	6.33	20050	87	129
57	1586	Q99497	PARK7_HUMAN	Parkinson disease 7	6.33	20050	53	90
58	291	Q9UHG3	PCYOX_HUMAN	Prenylcysteine oxidase 1	5.80	57003	36	109
59	397	P30101	PDIA3_HUMAN	Protein disulfide-isomerase A3	5.98	57146	36	107
60	1699	Q8N1T3	MYO1H_HUMAN	Unconventional myosin-Ih	9.19	120045	13	58
61	631	P19652	A1AG2_HUMAN	Alpha-1-acid glycoprotein 2	5.03	23873	38	80
62	597	P06733	ENOA_HUMAN	Alpha-enolase	7.01	41481	68	229
63	1634	P68871	HBB_HUMAN	Hemoglobin subunit beta	6.75	16102	95	149
64	340	Q6PCB0	VWA1_HUMAN	Von willebrand factor A domain containing protein 1	7.18	46889	34	67
65	764	P33176	KINH_HUMAN	Kinesin-1 heavy chain	6.12	110358	12	59
66	1637	P68871	HBB_HUMAN	Hemoglobin subunit beta	6.75	16102	84	176
67	334	P68871	HBB_HUMAN	Hemoglobin subunit beta	6.75	16102	84	155
68	421	P30101	PDIA3_HUMAN	Protein disulfide-isomerase A3	5.98	57146	52	119
69	1664	Q96N38	ZN714_HUMAN	Zinc finger protein	9.29	65979	30	64
70	324	P10109	ADX_HUMAN	Adrenodoxin, mitochondrial	5.51	19666	36	57
71	564	P08670	VIME_HUMAN	Vimentin	5.06	53676	70	349
72	345	Q8NB42	ZN527_HUMAN	Zinc finger protein 527	8.30	69001	30	58

73	315	P02675	FIBB_HUMAN	Fibrinogen beta chain	8.54	56577	52	106
74	344	O00159	MYO1C_HUMAN	Unconventional myosin-Ic	9.48	122503	18	58
75	292	Q07507	DERM_HUMAN	Dermatopotin	4.70	24559	33	59
76	972	P06733	ENOA_HUMAN	Alpha-enolase	7.01	47481	65	222
77	460	P01266	THYG_HUMAN	Thyroglobulin	5.40	311608	21	151
78	699	P01266	THYG_HUMAN	Thyroglobulin		311608	10	79
79	409	Q66GS9	CP135_HUMAN	Centrosomal protein of 135 kDa	5.87	133878	25	64
80	247	A8MUN3	YQ048_HUMAN	Putative uncharacterized protein ENSP00000381830	9.10	14538	15	58
81	1627	P15090	FABP4_HUMAN	Fatty acid-binding protein, adipocyte	6.59	14824	46	77
82	1259	P04406	G3P_HUMAN	Glyceraldehyde-3-phosphate dehydrogenase	8.57	36201	41	86
83	`308	Q13228	SBP1_HUMAN	Methanethiol oxidase	5.93	52928	39	99
84	1603	P30626	SORCN_HUMAN	Sorcin	5.32	21947	52	86
85	1465	Q1A5X7	WHAL1_HUMAN	Putative WASP homolog-associated protein with actin, membranes and microtubules-like protein 1	5.71	18193	42	58
86	328	Q96GX5	MASTL_HUMAN	Serin/threonine kinase greatwall	5.67	98398	28	59
87	1426	P02743	SAMP_HUMAN	Serum amyloid P-component	6.10	25485	38	125
88	969	P14550	AK1A1_HUMAN	Aldo-keto reductase family 1 member A1	6.32	36892	44	84

89	359	Q13228	SBP1_HUMAN	Methanethiol oxidase	5.93	52928	56	177
90	983	P40121	CAPG_HUMAN	Macrophage-capping protein	5.88	38779	37	66

[a] Spot number.

[b] Protein accession number for SWISSPROT Database.

[c] Theoretical isoelectric point.

[d] Theoretical relative mass.

[e] MASCOT score

Figure S1: 2D DIGE numbered spots specify proteins with differential abundance (fold-change ≥ 1.5, P ≤ 0.05) that were identified using MALDI-TOF mass spectrometry comparing tissue samples from BDG and control states. pI stands for isoelectric point.

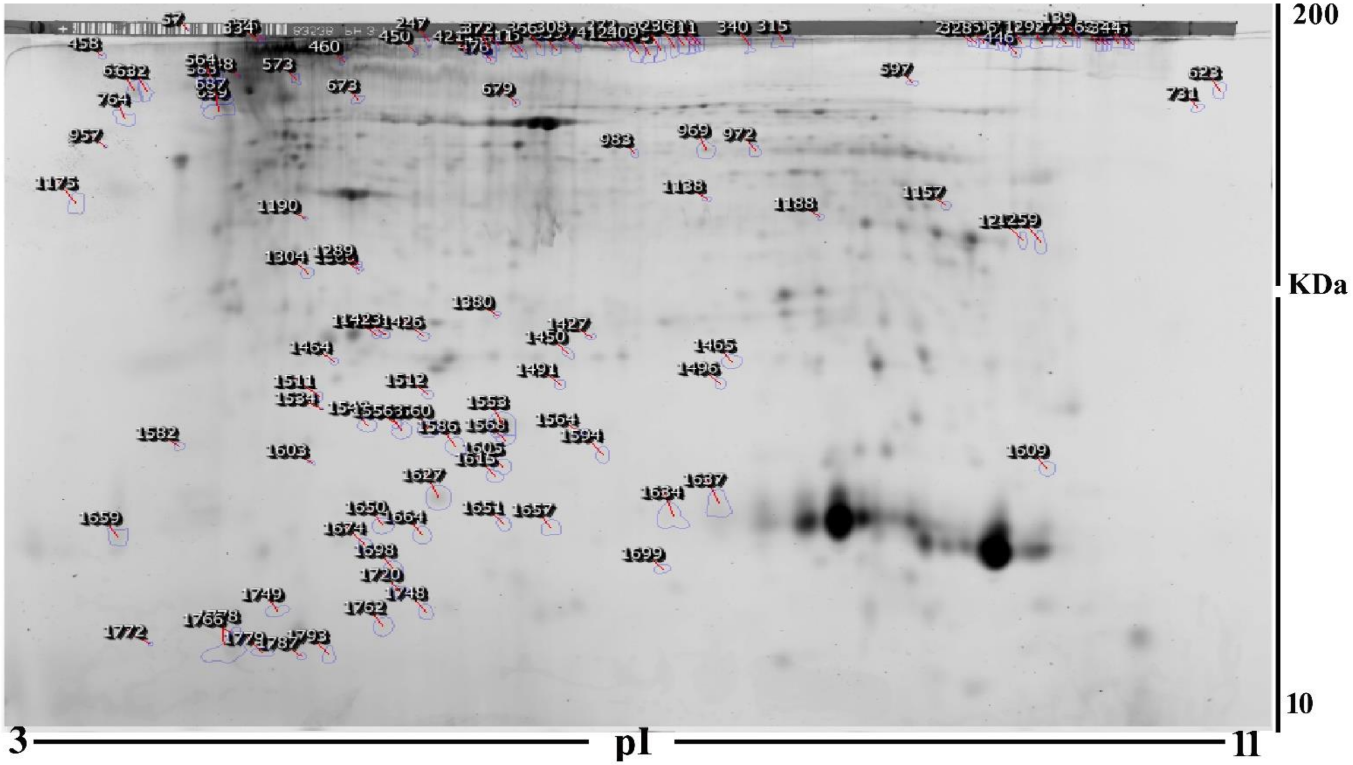

Figure S2: Pathways and canonical pathways identified in the IPA functional analysis.

Top Canonical Pathways

Name	p-value	Overlap
Acute Phase Response Signaling	4.08E-07	3.9 % 7/180
NRF2-mediated Oxidative Stress Response	5.68E-07	3.7 % 7/189
Clathrin-mediated Endocytosis Signaling	6.54E-07	3.6 % 7/193
Caveolar-mediated Endocytosis Signaling	3.84E-05	5.5 % 4/73
Glycolysis I	4.09E-05	11.5 % 3/26

Top Networks

ID	Associated Network Functions	Score
1	Endocrine System Disorders, Organismal Injury and Abnormalities, Cancer	48
2	Cellular Assembly and Organization, Cellular Development, Cellular Growth and Proliferation	31
3	Cancer, Immunological Disease, Organismal Injury and Abnormalities	23
4	Cancer, Gastrointestinal Disease, Hepatic System Disease	16
